# Orofacial Herpes Zoster Infection in Dental practice: A Case Report

**DOI:** 10.31729/jnma.5310

**Published:** 2020-11-30

**Authors:** Deepa Gurung, Ujjwal Joshi, Bikash Chaudhary

**Affiliations:** 1 Department of Oral Medicine and Radiology, Kathmandu Medical College and Teaching Hospital, Duwakot, Bhaktapur, Nepal; 2 Department of Oral and Maxillofacial Surgery, Kathmandu Medical College and Teaching Hospital, Duwakot, Bhaktapur, Nepal

**Keywords:** *herpes zoster*, *shingles*, *trigeminal nerve*, *varicella-zoster*

## Abstract

Herpes zoster infection, commonly known as Shingles, is caused by reactivation of the Varicella-Zoster virus which may have remained latent in the dorsal root ganglia. It is characterized by prodromal symptoms of unilateral deep aching, burning pain followed by a maculopapular rash, vesicular eruptions, ulcers, and scab formations over the affected nerve distribution. The ophthalmic branch of the trigeminal nerve is more commonly involved in herpes zoster infection than maxillary and mandibular branches; in particular, the maxillary involvement is rare. This is a case report of herpes zoster infection in a 65-years-old male patient involving the maxillary division of the trigeminal nerve. This case highlights the importance of early diagnosis and prompt use of antivirals in managing orofacial infection in dental practice.

## INTRODUCTION

Varicella-Zoster virus (VZV) is a member of the herpes family that may remain latent in the spinal and cranial sensory ganglia after the primary infection, which upon reactivation causes Herpes Zoster infection, commonly known as Shingles.^1^ Among cranial nerves, the ophthalmic division of the trigeminal nerve is most commonly affected^2^ while maxillary and mandibular branches are rarely involved, which shows oral manifestations.^3,4^ Reactivation is more common and severe as age advances as well as in immunocompromised patients such as in patients with HIV.^5^

## CASE REPORT

**Figure 1:**
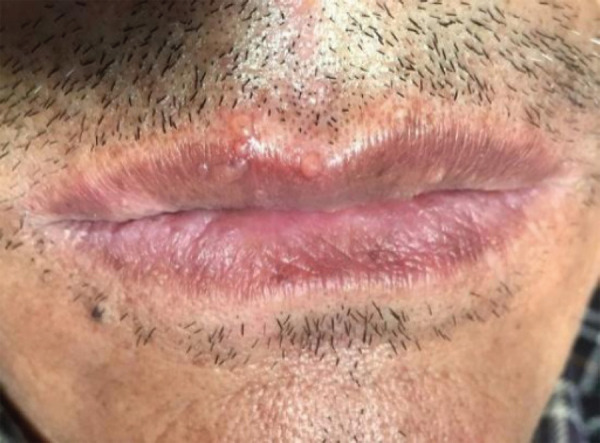
Few crops of vesicles on the right side of the upper lip region involving vermilion border (first visit).

A 65-years old male patient came to the Department of Oral Medicine and Radiology with the chief complaint of deep aching and burning pain on the right side of the face region for the last two days. On clinical examination, few crops of vesicles were present on the right side of his lip region involving the vermilion border (Figure 1) and a single vesicle was present intraorally on the right side of the palate region. Past medical history was insignificant.

Based on the history of a burning sensation on the right side of the maxillary dermatome region with a unilateral distribution of vesicles, it was provisionally diagnosed as herpes zoster with the involvement of the maxillary branch of the trigeminal nerve. The patient was kept under Tab. Acyclovir 800mg five times daily for seven days and recalled for follow-up after a week.

**Figure 2 (A,B,C):**
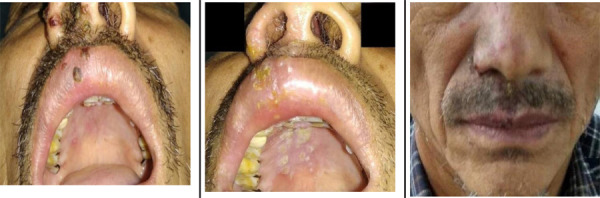
Scabs formations on the right side of the upper lip, ala of nose region and healed ulcerated lesions with erythema on right side of the palatal region seen on the eighth day (third visit) (A), Vesiculo-ulcerated lesions present on the right side of ala, columella of the nose, upper lip region with mild swelling and intraoral multiple ulcerated lesions restricted on the right side of the hard palate (second visit) (B), Scabs formation seen on the right side of the ala of nose and right upper lip region (third visit) (C).

However, the patient returned to the department after two days with hyperesthesia on the right side of his face causing him difficulty in performing daily chores as well as falling asleep. The patient also presented with multiple vesiculo-ulcerative lesions on the right side of the ala of the nose, columella of the nose, and the upper lip, involving the vermilion border with mild swelling of the upper lip (Figure 2A). Intraorally, multiple ulcers were noted on the right side of the hard palate region which was restricted to the midline. After a thorough history, it was revealed that the patient had not taken the full daily dose of the prescribed antiviral medication (Acyclovir) and rather took painkillers from a nearby pharmacy that did not subside his pain. The patient was further advised for some routine laboratory investigations such as complete blood count (CBC), random blood glucose test, CRP (C reactive protein) test, and enzyme-linked immunoassay (ELISA) test to rule out any asymptomatic human immunodeficiency virus (HIV).

**Figure 3:**
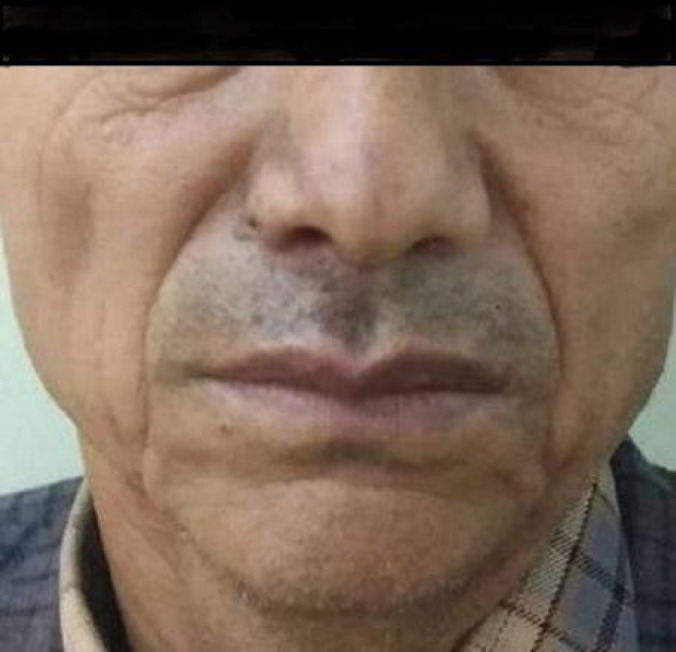
Complete resolution of extra-oral ulceration and scab lesions are seen on the 15^th^ day (fourth visit).

On day eight, the patient came with his laboratory reports which showed an increased erythrocyte sedimentation rate of 32mm/hr with all other investigations such as CBC, random glucose test, and CRP test results within the normal limits. ELISA test for HIV was negative. On examination, multiple scab formations were noted extra-orally (Figure 2B,2C).and much-healed ulcers intraorally. According to the patient, there was a marked reduction in pain and the burning sensation. Complete resolution of extraoral ulceration and scab lesions are seen on the 15th day (Figure 3). Nonetheless, the patient was made aware of the chances of recurrence of similar or worsening pain even after the resolution of lesions and was recalled for a follow-up visit after a week.

## DISCUSSION

Reactivation of VZV causes Herpes zoster or Shingles, in which involvement of maxillary and mandibular divisions of the trigeminal nerve show orofacial manifestation.^4^ Although data about HZI is limited in Nepal, few hospital-based studies have found the prevalence of maxillary branch involvement to be around 1-2% among all HZI patients.^6,7^ When the maxillary branch is involved, it can affect the cheek, ala of nose, upper and lower eyelids, upper gingiva,palate, tonsils, and nasopharynx.^2^ The characteristic unilateral pain with a burning sensation and vesicles restricted to the involved dermatome usually confirm the diagnosis.^4,5^ In our case, even though the initial lesions were very mild with few crops of vesicles on the right side of the lip region and a single vesicle on the right side of the hard palate region, their unilateral distribution with deep intense pain and a burning sensation on maxillary dermatome was suggestive of maxillary branch involvement of herpes zoster. However, a difficulty can arise in diagnosis when the pain is felt along the course of the nerve without any lesions, known as zoster sine herpete.^8^ Various investigations can be carried out for the confirmation of diagnosis, especially in patients with atypical presentations of HZI. Varicella-Zoster virus DNA polymerase chain reaction is often considered as the “gold standard” of diagnosis in herpes zoster.^8^ Reactivation of HZI is more common in older age as well as in immunocompromised patients such as HIV,^5^ therefore, ELISA test should also be carried out.

Management of herpes zoster includes the use of antiviral drugs such as systemic acyclovir 800mg five times daily for 7-10 days or use of famciclovir500mg three times daily or valacyclovir 1000mg three times daily for seven days.^5^ Postherpetic neuralgia(PHN) is the most important complication of herpes zoster in which the pain lingers even after a month of onset of rash.^9^ Literature review shows that additional use of corticosteroids in conjunction with antiviral drugs can be effectivein managing acute painin herpes zoster; however, the studies are inconclusive regarding its effectiveness in preventing PHN.^8,9^ Besides PHN, various other complications such as scarring and disfigurement, encephalitis, and even permanent loss of vision can also occur.^10^ Therefore, early diagnosis of lesions and the prompt use of antivirals can help manage the orofacial herpes zoster infection in dental practice.
